# Evaluating the Impact of Weekly Physical Performance Reports on Daily Physical Activity and Symptoms Among Cancer Survivors: Secondary Analysis of a Randomized Controlled Trial

**DOI:** 10.3390/cancers17172850

**Published:** 2025-08-30

**Authors:** Mingfang Li, Chitchanok Benjasirisan, Jingyu Zhang, Jennifer Yeong-Shin Sheng, Junxin Li, Sarah Allgood, Michael Carducci, Johannes Thrul, Nada Lukkahatai

**Affiliations:** 1 School of Nursing, Sun Yat-sen University, Guangzhou 510000, China; fang6@hku.hk; 2 School of Nursing, Li Ka Shing Faculty of Medicine, University of Hong Kong, Hong Kong SAR, China; 3 School of Nursing, Johns Hopkins University, Baltimore, MD 21205, USA; cbenjas1@jhmi.edu (C.B.); jzhan290@jhu.edu (J.Z.); junxin.li@jhu.edu (J.L.); sallgoo1@jhmi.edu (S.A.); 4 Johns Hopkins School of Medicine, Sidney Kimmel Comprehensive Cancer Center, Baltimore, MD 21231, USA; jsheng7@jhmi.edu (J.Y.-S.S.); carducci@jhmi.edu (M.C.); 5 Bloomberg School of Public Health, Johns Hopkins University, Baltimore, MD 21205, USA; jthrul@jhu.edu

**Keywords:** cancer, ecological momentary assessment, physical activity, symptoms, mHealth

## Abstract

**Background/Objectives:** Many cancer survivors continue to experience persistent symptoms such as pain, fatigue, and depression. Exercise and increasing physical activity (PA) are recommended as methods that can help alleviate these symptoms. However, maintaining regular exercise can be difficult due to the challenge of maintaining motivation. This secondary analysis uses data from a randomized controlled pilot study that examines the feasibility of the technology-enhanced combined exercise and other nonpharmacological interventions, such as the TEHEplus program, in managing symptoms. The program used mobile technologies and sent weekly physical performance reports to maintain engagement. In this study, we aimed to examine the impact of weekly physical performance reports on daily symptoms and PA and explored characteristics as moderators. **Methods:** This secondary analysis included participants who completed the 12-week TEHEplus program. Daily data of steps (PA), sleep duration, and symptoms (rated on a 4-point scale) were collected for 84 days. Mixed-effects and lagged regression models were used to assess the impact of weekly physical performance reports and the moderating effects of baseline characteristics. **Results:** Seventy-seven survivors of solid tumor cancer (mean age, 59.79, *SD* = 12.17) were analyzed. During this 84-day period, participants reported a reduction in low energy (*B* = −0.003, *p* = 0.004). Step counts increased by an average of 141 steps immediately after each weekly report (*B* = 140.857, *p* = 0.027). An increase of 5000 steps on the prior day was related to decreasing pain (*B* = −0.047, *p* < 0.001) and fatigue/low energy (*B* = −0.082, *p* < 0.001). Two additional hours of prior-night sleep worsened pain (*B* = 0.029, *p* = 0.001) and fatigue/low energy (*B* = 0.027, *p* = 0.002). Gender, employment, race, caregiver, and type of treatments moderate the relationship between daily PA, sleep (*B* = −0.131~0.078, *p_s_* < 0.05). **Conclusions:** Weekly physical performance reports promoted PA, which possibly led to self-reported improvement of symptoms. Higher PA was associated with better self-report symptoms, but longer sleep duration was associated with worsened symptoms, suggesting the need for future studies. The moderating effects suggested that tailoring interventions to individual profiles may enhance the program’s effectiveness.

## Simple Summary

Cancer survivors often continue to deal with symptoms such as tiredness, pain, and difficulty sleeping that affect their daily lives. Increasing physical activity was recommended as a strategy to help manage these symptoms. However, this recommendation can be challenging to follow because survivors may struggle to stay motivated and maintain regular activity. Our study tested whether a simple weekly report about overall steps, sleep, and symptoms could help survivors stay on track. Over 12 weeks, 77 participants used a phone app and fitness tracker, and the weekly reports encouraged them to walk more, which in turn was linked to feeling less pain and fatigue the next day. Over time, participants also reported feeling less tired. The longer sleep was unexpectedly related to worse pain and low energy. The overall results suggest that regular feedback can motivate survivors to be more active and improve their daily lives. Because cancer survivors may face different challenges depending on their health, background, and treatment history, personalized support is needed to make physical activity a realistic and sustainable option.

## 1. Introduction

Advancements in screening and treatment have significantly improved survival rates, leading to a growing population of cancer survivors who face persistent physical and psychosocial challenges, such as pain, fatigue, and sleep disturbances, which can severely impact quality of life [1,2]. Many cancer patients experience substantial symptom burden post-treatment, including moderate to severe pain (38%), low energy, and sleep issues, affecting nearly 60% globally [3,4,5,6]. Exercise interventions are widely recognized as beneficial for improving physical and psychological well-being, particularly for cancer survivors who face ongoing challenges such as fatigue, pain, and mood disturbances [7,8].

One of the significant challenges in most exercise programs is the lack of compliance and sustainability over time. Without ongoing support, individuals may struggle to maintain their exercise routines, leading to declines in physical activity (PA) and diminished health benefits [9]. Evidence suggests that reports on physical performance may enhance motivation through fostering autonomy, competence, and a sense of relatedness, which are crucial for sustaining engagement in health-promoting behaviors [10,11]. Feedback, specifically a weekly physical performance report, may reinforce self-monitoring, facilitate timely adjustments in behavior, and help individuals remain aligned with their exercise goals [12,13]. However, other research indicates that feedback may not always produce the intended positive effects on behavior. Overly frequent or intrusive feedback can lead to feedback fatigue and reduce motivation over time [14]. Moreover, feedback that focuses solely on quantitative metrics such as step counts may shift attention away from intrinsic motivation and enjoyment, making exercise seem obligatory rather than self-directed, which can hinder long-term adherence [15].

A common limitation of prior exercise and symptom management interventions is the reliance on recall-based self-reports, which are subject to bias and inaccuracies [16,17]. Participants often face challenges in accurately remembering and reporting the intensity of symptoms and quantity of their PA sessions, leading to discrepancies between perceived and actual behaviors [18,19]. This recall bias is particularly problematic for managing symptoms, as self-reports may overlook moment-to-moment pain, fatigue, and motivation fluctuations affecting exercise adherence and outcomes.

Ecological Momentary Assessment (EMA) addresses these issues by collecting data in real-time within natural settings, minimizing recall bias, and providing a more accurate, immediate picture of behaviors and symptom changes [20,21,22]. It is known that both symptoms and PA levels can fluctuate considerably from day to day. Relying on retrospective reports or periodic clinic visits may obscure important change patterns. Measuring these dynamics through daily EMA reporting can increase the understanding of how interventions change activity and symptom experiences in real-world settings. Moreover, integrating daily data into a structured weekly physical performance report may provide an opportunity for survivors to reflect on their progress, identify barriers, and adjust behaviors, potentially enhancing self-management and adherence to healthy activity routines. However, little is known about how weekly physical performance reports impact daily symptoms or activity levels.

In the parent study, Lukkahatai and colleagues [23] primarily aimed to evaluate the feasibility of a personalized, non-pharmacological intervention (TEHEplus) for symptom management in cancer survivors. The intervention used mobile technologies, including a physical activity tracker and a smartphone application, to monitor daily steps, sleep, and symptom severity. As part of the protocol, participants received weekly physical performance reports summarizing their activity and symptom trends. However, at the time, the potential impact of these weekly feedback reports on participants’ daily symptoms and physical activity was not anticipated or systematically examined.

**Figure 1:**
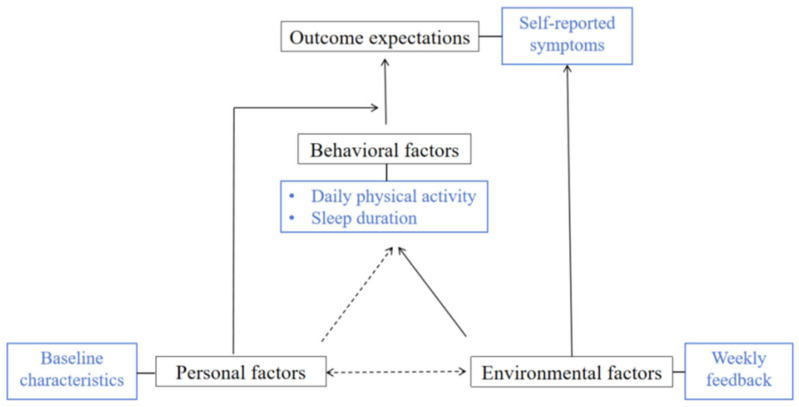
Conceptual Framework. Note: The solid lines represent the relationship we explored in this study; The dotted lines represent the relationships explained by Bandura’s Social Cognitive Theory but were not explored in this study.

This study is a secondary analysis of Lukkahatai and colleagues’ TEHEplus study [23], focusing on the impact of weekly performance summaries on daily changes in step count, sleep duration, and symptoms among cancer survivors. The analysis is guided by Bandura’s Social Cognitive Theory, which highlights the interactions between personal factors, environmental influences, and behaviors in generating lasting changes (see Figure 1) [10,13,24,25]. This framework emphasizes the role of self-efficacy, self-monitoring, and feedback in promoting sustained behavioral changes, suggesting that individuals are more likely to engage in health-promoting activities when they feel capable, receive ongoing feedback, and can track their progress.

To address this, we analyzed with three main objectives: (1) to examine the effect of weekly physical performance reports within a 12-week wearable device-based behavioral program on daily PA (step counts), sleep duration, and self-reported symptoms (pain, low energy, sleep difficulty, and mood disturbances); (2) to explore how the relationship between changes in PA, sleep duration and symptoms reports; and (3) to examine baseline factors may influence the relationships.

## 2. Method

This study is a secondary analysis of data from a five-arm, parallel-group, pilot randomized controlled trial (RCT), registered at ClinicalTrials.gov (NCT03576274).

### 2.1. Parent Trial Overview

In the parent study, adult survivors of solid tumors were recruited from a cancer center in Baltimore, Maryland, between October 2019 and September 2024. Eligibility criteria required participants to report fatigue of three or higher on a 0–10 scale in the past week, own a smartphone, and be able to walk continuously for at least six minutes with moderate exertion, defined as a Borg Rating of Perceived Exertion score of six or below. Exclusion criteria included active infection, severe psychiatric conditions such as uncontrolled anxiety or depression with suicidal ideation, and other contraindications such as unstable cardiopulmonary disease or severe anemia. The study was approved by the Johns Hopkins Medicine Institutional Review Board (IRB# 00154198).

A total of 110 participants were randomized in a 1:1:1:1:1 ratio to one of five groups: Usual Care Control, Acupressure, Technology-Enhanced Home Exercise (TEHE), TEHE combined with Acupressure, or TEHE combined with a Mindfulness-Based Intervention. Block randomization stratified by age (<60 vs. ≥60) and sex (male vs. female) was used to ensure balance across groups. All participants received a physical activity tracker and access to the TEHEplus smartphone application (developed by the parent study research team) to record daily symptoms and activity and to receive weekly reports summarizing steps, sleep duration, and symptom trends. Intervention group participants additionally received training and support for their assigned therapy. Both participants and study staff were aware of group assignments, but outcome assessments followed standardized procedures. The primary outcomes were feasibility measures (recruitment, adherence, and completion rates) and preliminary effects on fatigue and physical function assessed at baseline and after the 12-week intervention. Secondary outcomes included cancer-related symptoms, functional outcomes, and serum brain-derived neurotrophic factor (BDNF) levels. The trial demonstrated acceptable feasibility, with a retention rate of 71 percent and adherence of 80 percent in the intervention groups. Preliminary findings suggested that combined interventions improved fatigue and physical function compared with usual care, with additional trends indicating broader potential benefits.

### 2.2. Secondary Analysis Methods

#### 2.2.1. Design

This secondary analysis focused on participants who completed the 12-week intervention and provided sufficient app-based data for longitudinal modeling. Unlike the parent trial, which compared multiple intervention groups, we exclusively examined the effects of weekly physical performance reports that were delivered to all participants.

#### 2.2.2. Sample and Setting

Purposive sampling was used to select participants who completed the 12-week program (*n* = 77). Therefore, a prospective sample size calculation was not performed. A post hoc power analysis using G*Power 3.1.9.7 showed that with the sample size of 77, the study had 85% power to detect a medium effect (*α* = 0.05, *d* = 0.35).

#### 2.2.3. Data Source

Data for this secondary analysis study came from the secure cloud platform of both the TEHEplus smartphone application and the activity tracker used in the parent study. All participants used the TEHEplus smartphone application and a commercially available activity tracker to record their daily activity, sleep, and symptom severity.

#### 2.2.4. Measures and Outcomes

**Figure 2:**
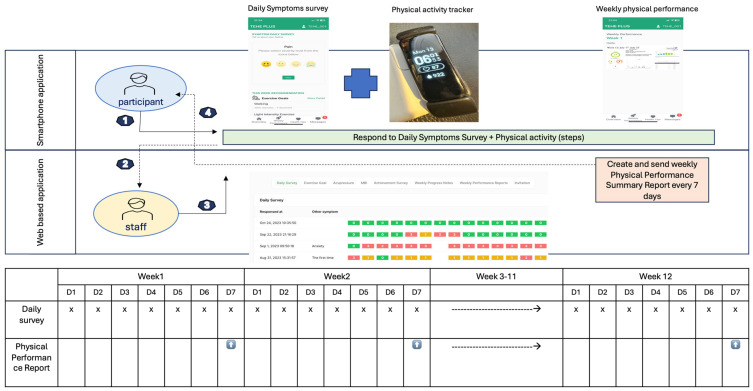
Flowchart of Data Collection and Reporting Process for the TEHEplus Program.

*Weekly Physical Performance Reports*: Each week, research staff compiled participants’ daily entries from the TEHEplus app and tracker (symptoms, step counts, sleep duration) from the previous 7 days. The data were summarized into weekly physical performance reports and delivered to participants through the app. Participants received a performance feedback report via the app each week summarizing steps, sleep, and symptom trends. We selected a weekly frequency to provide consistent reinforcement while minimizing participant burden, which is consistent with prior mHealth interventions in cancer survivors [26,27,28,29]. Our analysis used these weekly reports as the intervention exposure of interest (see Figure 2).

*Demographic and clinical data*: Age, race, ethnicity, estimated household income, educational attainment, family caregiver presence, employment status, marital status, cancer type, adjunct treatments (e.g., chemotherapy, radiation, hormonal therapy, immunotherapy), and comorbidities were collected at baseline.

*Symptom Assessment*: Baseline severity of pain, fatigue, sleep disturbance, and mood was assessed using a 0–10 Likert scale (0 = no symptoms, 10 = worst imaginable symptoms). Ongoing daily symptoms (pain, low energy, sleep difficulties, mood disturbances) were reported on the TEHEplus app using a 4-point scale (0 = none; 1 = mild; 2 = moderate; 3 = severe) and securely stored on a cloud-based platform. The MD Anderson Symptom Inventory, a validated 0–10 scale, was used for baseline symptom assessment [30].

*Physical Activity and Sleep*: Daily step counts (steps/day) and sleep duration (minutes/night) were continuously measured using the commercially available PA tracker, Fitbit Charge (Fitbit Inc., San Francisco, CA, USA). Data were recorded in the device app and exported for analysis. This commercial PA tracker has demonstrated acceptable validity for physical activity metrics, with intraclass correlation coefficients of 0.81–0.96 compared to gold-standard measurements [31].

### 2.3. Data Analysis

Analyses were performed using SPSS 25.0 (IBM Corp., Armonk, NY, USA) and Stata 15.0 (StataCorp LLC, College Station, TX, USA). Descriptive statistics for participants’ demographic and clinical characteristics included means and standard deviations (*SD*) for continuous variables and frequencies and percentages for categorical variables. Missing data was handled using multiple imputation techniques. A mixed-effects model was used to examine the daily trajectories of step counts, sleep duration, and symptom severity over time, the impact of weekly physical performance reports on daily changes in these outcomes within each week, and how these effects evolved across the 84-day study period (time × physical performance reports interaction), with individual variability accounted for as random effects. A lagged regression model explored relationships between changes in PA, sleep duration, and symptom severity, creating two lagged variables—step_lag and sleep_lag—to assess the delayed effects of the previous day’s step counts and sleep duration on the following day’s symptoms. Step counts were standardized to units of 5000 steps, and sleep duration was converted to units of 2 h for interpretability, where each unit increase in step_lag represented the effect of an additional 5000 steps, and each unit increase in sleep_lag reflected the effect of an additional 2 h of sleep. Interaction terms were added to the regression model to investigate moderating effects of baseline characteristics, such as gender, age, employment status, and baseline core symptom severity, retaining only statistically significant relationships to avoid overfitting and maintain rigor. Some categories were reclassified during the moderation analysis to address variability in certain baseline characteristics and enhance interpretability.

## 3. Results

### 3.1. Sample Characteristics

A total of 77 participants were included in this analysis. The mean age was 59.8 years (*SD* = 12.2), and 58.4% identified as male. The majority identified as White (87.0%) and non-Hispanic or Latino (88.3%), with most reporting a college or postgraduate education (80.5%) and an estimated annual household income of $75,000 or greater (88.3%). Nearly half were employed (49.4%), and most identified themselves as their primary caregiver (89.6%). The majority were married or in a domestic relationship (83.1%). Prostate cancer was the most common diagnosis (33.8%), followed by breast cancer (19.5%). Most participants reported at least one comorbidity (97.4%), while a smaller proportion were undergoing chemotherapy (11.7%), radiation (3.9%), hormonal therapy (20.8%), or immunotherapy (20.8%). Baseline mean step count was 6377 steps/day (*SD* = 4374), mean sleep duration was 5.88 h/night (*SD* = 2.87), and mean baseline symptom scores were low to moderate for pain (2.32), low energy (4.61), sleep difficulty (3.74), and mood disturbances (2.64). Additional demographic and clinical characteristics are presented in Table 1.

### 3.2. Effect of Weekly Physical Performance Reports

In mixed-effects models, there was a significant overall time effect for self-reported low energy, with a gradual decrease over the 12-week period (*B* = −0.003, *p* = 0.004). Examining the effect of weekly reports of physical performance, a significant positive impact was observed on daily step counts, with an average increase of approximately 141 steps following each weekly report (*B* = 140.86, *p* = 0.027). No significant weekly report effects were found for other outcomes, including sleep duration, pain, low energy, sleep difficulty, or mood disturbances (all *p_s_* > 0.05). (see Table 2).

### 3.3. Relationships Between Changes in Daily Physical Activity, Sleep, and Symptoms

Regression analyses revealed that increases in step count from the previous day were significantly associated with reductions in both pain (*B* = −0.047, *p* < 0.001) and low energy (*B* = −0.082, *p* < 0.001) the following day. Specifically, for each 5000-step increase, participants reported significantly less pain and fatigue the next day. Conversely, longer sleep duration the previous night was associated with higher pain (*B* = 0.029, *p* = 0.001) and lower energy scores (*B* = 0.027, *p* = 0.002) the next day; each additional 2 h of sleep predicted worse self-reported pain and lower energy. No significant associations were observed between step or sleep changes and other symptoms (all *p_s_* > 0.05) (see Table 3).

### 3.4. Effect of Baseline Characteristics on Relationships Among Physical Activity, Sleep, and Symptoms

Further regression analyses investigated moderation by baseline characteristics. For pain, the reduction associated with increased step count was greater in non-employed participants (*p* = 0.020) and females (*p* = 0.041). Participants cared for by significant others (as opposed to self-care) benefited less in terms of pain reduction from increased steps (*p* = 0.011). Higher baseline step counts were associated with diminished benefits of increased steps for pain reduction (*p* = 0.002). For low energy, non-employed participants experienced greater reductions in fatigue with increased steps (*p* = 0.030), while those with higher incomes had a weaker improvement in energy levels (*p* = 0.002). Regarding sleep, the relationship between increased sleep and low energy was stronger among participants with significant others as caregivers (*p* = 0.015), those receiving chemotherapy (*p* = 0.001) or hormonal therapy (*p* = 0.026), and participants of “Other” race (*p* = 0.043). Female participants showed a weaker association between increased sleep and energy improvement compared to males (*p* = 0.003) (see Table 4).

## 4. Discussion

This study contributes evidence on the influence of weekly physical performance reports on daily physical activity and symptoms among cancer survivors engaged in a personalized non-pharmacological intervention. Using ecological momentary assessment (EMA), we offer insights into the day-to-day relationships between step counts, sleep duration, and cancer-related symptoms. Our findings indicate that weekly feedback reports were associated with increased daily step counts, supporting the use of regular, structured feedback as a means of promoting physical activity. This result is consistent with prior research and Bandura’s Social Cognitive Theory, which suggested that feedback mechanisms can effectively motivate increased physical activity in various populations, including cancer survivors [12,13]. However, the absence of a sustained or cumulative effect over the intervention period may suggest that repeated exposure to uniform feedback can lead to diminishing motivational returns [14,15]. This highlights the importance of refining feedback strategies, potentially by varying content, format, or frequency, to maintain engagement and support long-term behavior change.

No significant influence of weekly feedback was observed on sleep duration or cancer-related symptoms such as pain, fatigue, and mood disturbances. It is possible that while step count is a straightforward behavioral target with concrete goals, sleep and symptom improvement are influenced by a complex interplay of physical, psychological, and medical factors [32,33,34]. Thus, feedback on PA alone may not be sufficient to produce meaningful changes in sleep or other symptoms. From a social cognitive perspective, this may reflect the task-specific nature of self-efficacy; participants may have felt more capable of modifying a concrete behavior like walking than influencing more complex, multifactorial symptoms such as sleep disturbance or fatigue. Future feedback-based interventions may benefit from using more engaging and tailored content to better target specific symptoms.

For the time main effect, our result showed a decreasing trend of self-reported low energy score across 84 consecutive days, suggesting that the intervention may have contributed to improvements in perception of energy over time. Similar results were also found in the previous exercise-based interventions for fatigue improvement among the cancer population [8,35]. It might be explained by the fact that regular exposure to exercise guidance could enhance patients’ fatigue management strategies, helping them avoid the gradual fatigue reduction.

The analysis of day-to-day changes revealed that increased physical activity on one day was associated with reduced pain and improved energy on the following day. These findings supported existing evidence that physical activity can have both immediate and delayed benefits for symptom relief, potentially via endorphin release, anti-inflammatory effects, and enhanced energy metabolism [36,37]. Moreover, the improvement of cellular energy metabolism helps relieve the cancer-related fatigue [38,39]. This reminds us that medical staff should take more action to encourage cancer survivors’ daily physical activity for better symptom relief.

An unexpected result was that the longer sleep duration (more than 7 h of sleep/night) was associated with severe pain and self-reported low energy. This finding seems to contradict the common assumptions that adequate sleep (usually more than 7 h/night) can alleviate cancer-related symptoms [40,41]. Prior research suggested a U-shaped relationship between sleep duration and health outcomes in cancer survivors, where both insufficient and excessive sleep are related to worse symptoms and prognosis [42,43]. Several plausible mechanisms may help explain this finding. First, survivors with more severe pain or fatigue may spend more time in bed or sleep longer as a coping mechanism, rather than sleep itself worsening symptoms. This explanation is consistent with a longitudinal finding that prolonged sleep (9 h or more/night) among cancer survivors often suggests greater disease burden, poorer prognosis, and more severe symptoms [44]. Second, prolonged time in bed can lead to stiffness or discomfort, which may increase pain and fatigue. Finally, oversleeping may be related to hypersomnia or circadian rhythm disruptions, which can reduce alertness and worsen fatigue [45]. These findings suggested the complexity of the relationship between sleep and symptom burden. Further research is needed to clarify the role of sleep duration in cancer survivorship.

Furthermore, our findings demonstrated the moderating role of baseline characteristics in the relationship between weekly physical performance reports and cancer-related symptoms. This corroborates Bandura’s Social Cognitive Theory that behavior change can be influenced by personal factors [24]. Special attention should be given to females, non-employed individuals, nonwhite participants, those undergoing chemotherapy or hormonal therapy, and individuals whose primary caregivers are significant others. Collectively, these results expand our understanding of how regular technology-enabled feedback can influence health behaviors and symptoms in cancer survivors, highlighting important directions for the design and personalization of future interventions.

Lastly, from a theoretical perspective, the findings of this study are consistent with Bandura’s Social Cognitive Theory (see Figure 1) [24]. Behavioral factors, such as patients’ daily physical activity and sleep duration, were associated with a person’s reported symptoms, while personal factors, including baseline characteristics, appeared to moderate these relationships. Environmental factors, represented by the weekly feedback, also played a role in shaping participants’ experiences. These patterns emphasize the interaction of behavioral, environmental, and personal influences suggested by Social Cognitive Theory. The weekly reports may have supported self-regulation and task-specific self-efficacy for walking, a behavior that is more concrete and controllable than multifactorial outcomes such as sleep or fatigue. These observations tentatively illustrate the principle of reciprocal determinism, suggesting that feedback-based strategies may be more effective when they account for the interplay of individual characteristics, behaviors, and environmental supports.

### Limitations

This study has several limitations that warrant consideration. First, the relatively small sample size may have limited the statistical power to detect more subtle associations and restricted the generalizability of our findings to broader or more diverse cancer survivor populations. Additionally, participants were recruited from a single cancer center and were predominantly White, higher-income, and well-educated, which further limits the external validity of our results and may not fully represent the experiences of more diverse or socioeconomically disadvantaged individuals. Second, all behavioral and symptom data were based on self-reported ecological momentary assessment (EMA), which, despite minimizing recall bias, remains subject to inaccuracies due to compliance issues, subjective interpretation of items, or social desirability bias. Device-based measures, such as step counts and sleep duration captured through commercially available activity trackers, are generally objective but can also be affected by technical errors, improper device use, or syncing problems.

Moreover, as a secondary, observational analysis, our study cannot establish causal relationships between weekly feedback and the outcomes of interest. Other unmeasured variables, such as changes in treatment regimen, psychosocial factors, or environmental influences, could have confounded our results. Furthermore, we examined the overall impact of weekly report delivery but did not differentiate between the specific behavioral intervention components assigned in the parent trial, limiting our ability to address potential interactions between feedback and individualized intervention strategies.

Additionally, the feedback reports provided to participants were standardized and did not adapt or diversify over time. The lack of dynamic or personalized feedback content may have reduced the sustained motivational impact on outcomes, particularly as the intervention progressed. Also, there was no assessment of participants’ actual engagement with the feedback reports. Future research should incorporate measures to evaluate participants’ engagement and digital health literacy, emphasizing the critical role these factors play in the effectiveness of feedback-based interventions. Despite these limitations, the study offers important insight into the role of technology-enabled feedback in cancer survivorship and identifies areas for future research and intervention refinement.

## 5. Conclusions

Weekly physical performance reports modestly increased daily physical activity but did not significantly impact sleep duration or cancer-related symptoms. Increased daily activity was associated with improved pain and self-reported energy level, while longer sleep duration unexpectedly predicted worse symptoms. Self-reported lack of energy showed a gradual improvement over time. These findings suggested that providing feedback may play a role in supporting physical activity and related symptom outcomes, which is consistent with Bandura’s Social Cognitive Theory. Baseline sociodemographic and clinical characteristics moderated these relationships, suggesting that feedback-based intervention may need to be adapted for subgroups of patients. Our findings suggested that subgroups, such as women, non-white individuals, the unemployed, and those undergoing treatment or relying on caregivers, may require additional support. Future studies are needed to refine how these approaches can best be integrated into survivorship care.

## Figures and Tables

**Table 1:** Demographic and clinical information of the participants (*n* = 77).

Variables	*n*	%
**Age, (mean ± SD)**	59.79 ± 12.17	
**Gender**		
Male	45	58.4
Female	32	41.6
**Race**		
White	67	87.0
Black or African American	4	5.2
Others	6	7.8
**Ethnicity**		
Hispanic or Latino	4	5.2
Not Hispanic or Latino	68	88.3
Unknown	5	6.5
**Estimated Annual Household Income**		
<$75,000	9	11.7
≥$75,000	68	88.3
**Education**		
≤High School Graduate	3	3.9
Some College	12	15.6
College/Postgraduate	62	80.5
**Primary Caregiver**		
Self	69	89.6
Significant others	8	10.4
**Employment**		
Employed	38	49.4
Non-employed	39	50.6
**Marital Status**		
Married/Domestic partnership	64	83.1
Not married	13	16.9
**Cancer Type**		
Breast cancer	15	19.5
Prostate cancer	26	33.8
Others	36	46.7
**Undergoing Chemotherapy**		
Yes	9	11.7
No	68	88.3
**Undergoing Radiation**		
Yes	3	3.9
No	74	96.1
**Undergoing Hormonal therapy**		
Yes	16	20.8
No	61	79.2
**Undergoing Immunotherapy**		
Yes	14	18.2
No	63	81.8
**Comorbidity**		
Yes	75	97.4
No	2	2.6
**Baseline average step counts/day (mean ± SD)**	6376.77 ± 4374.17	
**Baseline average sleep hour/day, (mean ± SD)**	5.88 ± 2.87	
**Baseline pain severity, (mean ± SD)**	2.32 ± 2.69	
**Baseline low energy severity, (mean ± SD)**	4.61 ± 2.27	
**Baseline sleep difficulty severity, (mean ± SD)**	3.74 ± 2.75	
**Baseline mood disturbances severity, (mean ± SD)**	2.64 ± 2.70	

**Table 2:** Mixed-Effects Model of the weekly physical performance reports effect on PA, sleep, and symptoms (*n* = 77).

Outcomes and Predictors			Coefficient (95% CI)	*p*
Physical activity		time ^a^	−4.327 (−15.9498, 7.2957)	0.466
		Reports ^b^	140.857 (16.1327, 265.5820)	0.027 *
		Reports × time ^c^	−2.034 (−4.6278, 0.5613)	0.125
Sleep		Time	−0.398 (−0.8378, 0.0427)	0.077
		Reports	1.109 (−3.6311, 5.8484)	0.647
		Reports × time	0.043 (−0.0557, 0.1410)	0.395
Symptoms	Pain	time	−0.002 (−0.0029, 0.0003)	0.113
		Reports	−0.002 (−0.0243, 0.0203)	0.856
		Reports × time	9.530 × 10^−6^ (−0.004, 0.0004)	0.960
	Low energy	time	−0.003 (−0.0047, −0.0009)	0.004 *
		Reports	0.002 (−0.0186, 0.0225)	0.850
		Reports × time	4.510 × 10^−5^ (−0.0003, 0.0004)	0.818
	Sleep difficulty	time	−0.002 (−0.0043, 0.0002)	0.074
		Reports	0.011 (−0.0111, 0.0340)	0.314
		Reports × time	−1.521 × 10^−5^ (−0.0006, 0.0003)	0.527
	Mood disturbances	time	−6.568 × 10^−4^ (−0.0020, 0.0007)	0.327
		Reports	−1.285 × 10^−4^ (−0.0165, 0.0163)	0.987
		Reports × time	−1.700 × 10^−5^ (−0.0004, 0.0003)	0.920

Note: ^a^: the daily change across the period of 84 consecutive days; ^b^: the impact of physical performance reports on daily changes within each week; ^c^: whether the effects of weekly physical performance reports changed with time (84 days). PA: physical activity; * *p* < 0.05.

**Table 3:** Effects of Lagged PA and Sleep on Symptoms (*n* = 77).

Outcomes		Predictors	Coefficient	SE	95% CI	*t*	*p*	*F*
Symptoms	Pain	Step_lag ^d^	−0.047 ***	0.011	(−0.07, −0.02)	−4.16	<0.001	15.13 (*p* < 0.001)
		Sleep_lag ^e^	0.029 **	0.009	(0.01, 0.05)	3.30	0.001	
	Low energy	Step_lag	−0.082 ***	0.012	(−0.11, −0.06)	−7.03	<0.001	32.04 (*p* < 0.001)
		Sleep_lag	0.027 **	0.009	(0.01, 0.04)	3.12	0.002	
	Sleep difficulty	Step_lag	−0.206	0.013	(−0.05, −0.01)	−1.62	0.106	2.18 (*p* = 0.114)
		Sleep_lag	0.117	0.010	(−0.01, −0.03)	1.18	0.240	
	Mood disturbances	Step_lag	−2.079 × 10^−4^	0.009	(−0.02, 0.19)	−0.02	0.982	0.10 (*p* = 0.901)
		Sleep_lag	0.003	0.007	(−0.01, 0.02)	0.48	0.633	

Note: ^d^: the relationship between an additional 5000 steps in the previous day and the symptoms on the next day; ^e^: the relationship between an additional 2 h of prior-day sleep duration and the symptoms on the following day. PA: physical activity; ** *p* < 0.01; *** *p* < 0.001.

**Table 4:** Effects of Baseline Characteristics (*n* = 77).

Symptoms	Moderators	Coefficient	SE	95% CI	*t*	*p*
Pain	Employment × Step_lag (reference: employed)					
	non-employed	−0.048	0.021	(−0.0890, −0.0078)	−2.34	0.020 *
	Gender × Step_lag (reference: male)					
	female	−0.039	0.019	(−0.0757, −0.0016)	−2.04	0.041 *
	Race × Step_lag (reference: white)					
	Black or African American	0.009	0.690	(−0.1263, 0.1441)	0.13	0.897
	Others	−0.086	0.044	(−0.1725, 0.0001)	−1.96	0.050 *
	Caregiver × Step_lag (reference: self)					
	Significant others	0.078	0.031	(0.1800, 0.1381)	2.55	0.011 *
	Step_baseline × Step_lag	−0.037	0.012	(−0.0600, −0.0132)	−3.06	0.002 **
Low energy	Employment × Step_lag (reference: employed)					
	non-employed	0.001	2.96 × 10^−4^	(0.0001, 0.0012)	2.16	0.030 *
	Gender × Sleep_lag (reference: male)					
	female	−0.001	3.08 × 10^−4^	(−0.0015, −0.0003)	−2.99	0.003 **
	Race × Sleep_lag (reference: white)					
	Black or African American	0.085	0.114	(−0.1388, 0.3080)	0.74	0.458
	Others	0.072	0.036	(0.0022, 0.1425)	2.02	0.043 *
	Caregiver × Sleep_lag (reference: self)					
	Significant others	0.059	0.024	(0.0114, 0.1059)	2.43	0.015 *
	Income × Step_lag (reference: <$75,000)					
	≥$75,000	−0.131	0.042	(−0.2140, −0.0476)	−3.08	0.002 **
	Chemotherapy × Sleep_lag	0.002	2.47 × 10^−4^	(0.0006, 0.0024)	3.32	0.001 **
	Hormonal therapy × Sleep_lag	0.001	3.81 × 10^−4^	(0.0001, 0.0016)	2.23	0.026 *

Note: * *p* < 0.05; ** *p* < 0.01.

## Data Availability

The study is reported according to the CONSORT 2025 checklist (Supplementary Table S1). The data that support the findings of this study are available from the corresponding author upon reasonable request.

## Supplementary Materials

The following supporting information can be downloaded at: https://www.mdpi.com/article/10.3390/cancers17172850/s1, Table S1. CONSORT 2025 checklist. Reference [46] is cited in the Supplementary Materials.
